# Sidecar Learning: a new e-learning platform for librarians

**DOI:** 10.5195/jmla.2020.1048

**Published:** 2020-10-01

**Authors:** Yvonne Mery

**Affiliations:** 1 ymery@arizona.edu, Associate Librarian, University of Arizona Libraries, University of Arizona, Tucson, AZ

## Abstract

This article describes how two librarians at the University of Arizona created a new e-learning tool, Sidecar Learning, and implemented tutorials aimed at health sciences students and researchers. Librarians at the University of Arizona have used these interactive tutorials to reach thousands of students. Sidecar Learning was created to provide librarians with a scalable means of teaching thousands of students how to use complicated library databases.

For more than ten years, librarians at the University of Arizona Libraries (UAL) used a locally developed learning tool, the Guide on the Side (GOTS), to create online, interactive tutorials. GOTS was an open source tool that won national awards and was widely used by librarians across the United States. Unfortunately, the UAL did not have the resources to support and further develop GOTS.

The author and a colleague who serves as the business librarian used GOTS to teach tens of thousands of students how to use different databases every year and did not want to see it go away. We also knew that many other libraries were using it and even more wanted to use it but could not use an open source software that required local hosting. Thus, we decided to pursue different ways that we could keep GOTS alive. After looking at several grants, we decided that to be able to sustain and enhance GOTS, we needed to develop a completely new product that did not depend on grant funding. This eventually led to the development of a new electric learning tool and business, Sidecar Learning. TechLaunch Arizona, the office at the University of Arizona that commercializes inventions from faculty and staff, worked with us to help create the company and funded the beta version of the tool.

Sidecar Learning tutorials are interactive guides that work with any web resource, including library databases. The platform is built on a SQL backend, Ruby on Rails, and React JavaScript. Sidecar Learning guides open up next to any website and guide users through that website. Depending on the type of web resource and safety protocol it uses, Sidecar Learning guides can open in one window with an iframe or two separate windows that open next to each other. Librarians can easily add directions, comprehension checks, images, and videos. This arrangement provides students with an authentic and interactive learning experience, leading to better retention of information and understanding of how to use a particular database.

In a pilot research study of twenty-eight undergraduate students, we found that students who completed a tutorial were able to use the Academic Search Ultimate database more effectively and efficiently than students who did not complete a tutorial (Figure 1). The most significant difference occurred for the searching task: the time it took a student to start a search, evaluate results, and select an article.

**Figure 1:**
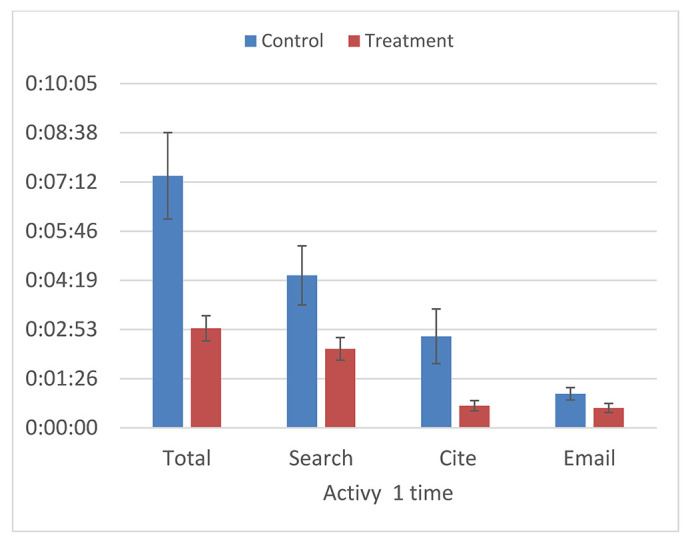
Time on task for control and treatment groups

UAL successfully implemented the Sidecar Platform in the spring semester, and over 8,000 students have completed tutorials to date. Along with librarians at the Arizona Health Sciences Library, we recently completed a series of PubMed tutorials for the roll out of its new interface (Figure 2). This tutorial

**Figure 2:**
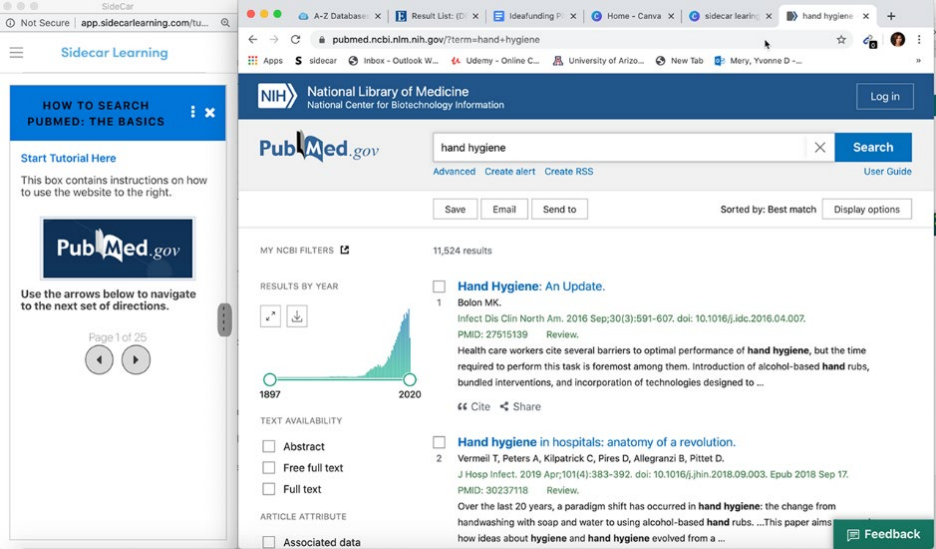
Sidecar Learning screenshot

This tutorial will be used by thousands of students across the United States and at global campuses.

The tutorial is made up of an introductory video and two Sidecar Learning guides. The first tutorial guides students through the main search features of PubMed, including how to add filters, how to access full text, and how to find similar articles. The second tutorial guides users through advanced search features including using Medical Subject Headings (MeSH), building searches from the history set, and finding clinical inquiries. We anticipate more than 4,000 students will take these tutorials each year. We will continue to use Sidecar Learning to develop tutorials for all our databases, starting with those that are most used by our students and faculty.

